# Evolution of the *Pseudomonas aeruginosa* mutational resistome in an international Cystic Fibrosis clone

**DOI:** 10.1038/s41598-017-05621-5

**Published:** 2017-07-17

**Authors:** Carla López-Causapé, Lea Mette Sommer, Gabriel Cabot, Rosa Rubio, Alain A. Ocampo-Sosa, Helle Krogh Johansen, Joan Figuerola, Rafael Cantón, Timothy J. Kidd, Soeren Molin, Antonio Oliver

**Affiliations:** 1 Servicio de Microbiología and Unidad de Investigación, Hospital Universitario Son Espases, Instituto de Investigación Sanitaria Islas Baleares (IdISBa), Palma de Mallorca, Spain; 2 Novo Nordisk Foundation Center for Biosustainability, The Technical University of Denmark, Lingby, Denmark; 3 Servicio de Microbiología, Hospital Universitario Marqués de Valdecilla, Instituto de Investigación Marqués de Valdecilla, Santander, Spain; 4 Servicio de Pediatría, Hospital Son Espases, Palma de Mallorca, Spain; 5 Servicio de Microbiología, Hospital Universitario Ramón y Cajal, Instituto Ramón y Cajal de Investigación Sanitaria (IRYCIS), Madrid, Spain; 6 School of Chemistry and Molecular Biosciences, The University of Queensland, Brisbane, QLD Australia; 7 Child Health Research Centre, The University of Queensland, Brisbane, QLD Australia

## Abstract

Emergence of epidemic clones and antibiotic resistance development compromises the management of *Pseudomonas aeruginosa* cystic fibrosis (CF) chronic respiratory infections. Whole genome sequencing (WGS) was used to decipher the phylogeny, interpatient dissemination, WGS mutator genotypes (mutome) and resistome of a widespread clone (CC274), in isolates from two highly-distant countries, Australia and Spain, covering an 18-year period. The coexistence of two divergent CC274 clonal lineages was revealed, but without evident geographical barrier; phylogenetic reconstructions and mutational resistome demonstrated the interpatient transmission of mutators. The extraordinary capacity of *P*. *aeruginosa* to develop resistance was evidenced by the emergence of mutations in >100 genes related to antibiotic resistance during the evolution of CC274, catalyzed by mutator phenotypes. While the presence of classical mutational resistance mechanisms was confirmed and correlated with resistance phenotypes, results also showed a major role of unexpected mutations. Among them, PBP3 mutations, shaping up β-lactam resistance, were noteworthy. A high selective pressure for *mexZ* mutations was evidenced, but we showed for the first time that high-level aminoglycoside resistance in CF is likely driven by mutations in *fusA1*/*fusA2*, coding for elongation factor G. Altogether, our results provide valuable information for understanding the evolution of the mutational resistome of CF *P*. *aeruginosa*.

## Introduction

*Pseudomonas aeruginosa* chronic respiratory infection (CRI) is the main driver of morbidity and mortality in patients suffering from cystic fibrosis (CF). The CF respiratory tract is a dynamic, heterogeneous, hostile, stressful and very challenging scenario for invading bacteria, but *P*. *aeruginosa* populations can overcome all these challenges and chronically persist in the CF lungs. Mechanisms underlying early acquisition of *P*. *aeruginosa* infection and the eventual establishment of CRI are complex and, many factors, related to the patient, the environment and the microorganism, are involved^1–3^.

The high versatility and adaptability observed for *P*. *aeruginosa* can be attributed to its complex and large genome (5–7 Mb), which includes an outstanding intrinsic antibiotic resistance machinery and a large proportion of regulatory genes (>8%). In comparison to other Gram-negative pathogens, *P*. *aeruginosa* exhibits a basal reduced susceptibility to many antibiotics, attributed to the production of an inducible AmpC cephalosporinase, the constitutive (MexAB-OprM) or inducible (MexXY) expression of efflux pumps, and the reduced permeability of its outer membrane. In addition, *P*. *aeruginosa* intrinsic resistance can be significantly enhanced by the acquisition of multiple mutations that alter the expression and/or function of diverse chromosomal genes^4–6^.

Early infection by *P*. *aeruginosa* in CF patients can be intermittent and usually different strains with distinct antibiotic susceptibility profiles are involved. However, according to the US CF Foundation Patient Registry, over 70% of CF patients are chronically colonized with *P*. *aeruginosa* by the age of 25 (Annual Report 2015); moreover, in up to 20% of them the involved strain is multidrug resistant (MDR). Although no single mutation can lead to an MDR profile, during CF CRI, *P*. *aeruginosa* is exposed to numerous and extended antimicrobial therapies which act as selective forces driving to the acquisition of a plethora of adaptive mutations that eventually lead to an enhanced antimicrobial resistance pattern. Moreover, this genetic adaptation is accelerated by the characteristic high prevalence of hypermutable strains (30–60%)^7–10^. Additionally, there is growing evidence suggesting that adaptation to the CF lungs may escape from the scale of the individual patients. Indeed, another remarkable and challenging issue in the CF setting is the existence of concerning *P*. *aeruginosa* epidemic strains, such as the Liverpool Epidemic Strain (LES-1), the Denmark Epidemic Strain (DK2) or the Australian Epidemic Strains (AES-1, AES-2 and AES-3), as these successful strains are able of infecting hundreds of CF patients even in different geographical locations and, indeed, in many cases exhibiting a MDR profile^11–13^. Therefore, CF CRI by widespread strains may provide a unique and exceptional opportunity to get insight into long-term evolutionary dynamics of *P*. *aeruginosa* mutational resistome.

Recent advances in sequencing technologies have made it possible to obtain the whole genome of bacterial pathogens. As mentioned above, *P*. *aeruginosa* CF CRI represent a unique chance to perform evolutionary studies and, accordingly, several works have been performed in this setting; however, most have focused their attention in pathoadaptive mutations^14^. Moreover, *P*. *aeruginosa* chronic infections are not limited to CF patients, being also frequently implicated in other chronic underlying diseases such as bronchiectasis and chronic obstructive pulmonary disease (COPD)^15^. Thus, an insight into the resistome evolution during CF CRI could be of great benefit not only for individual patients but also for developing new drugs and new treatment strategies.

**Figure 1:**
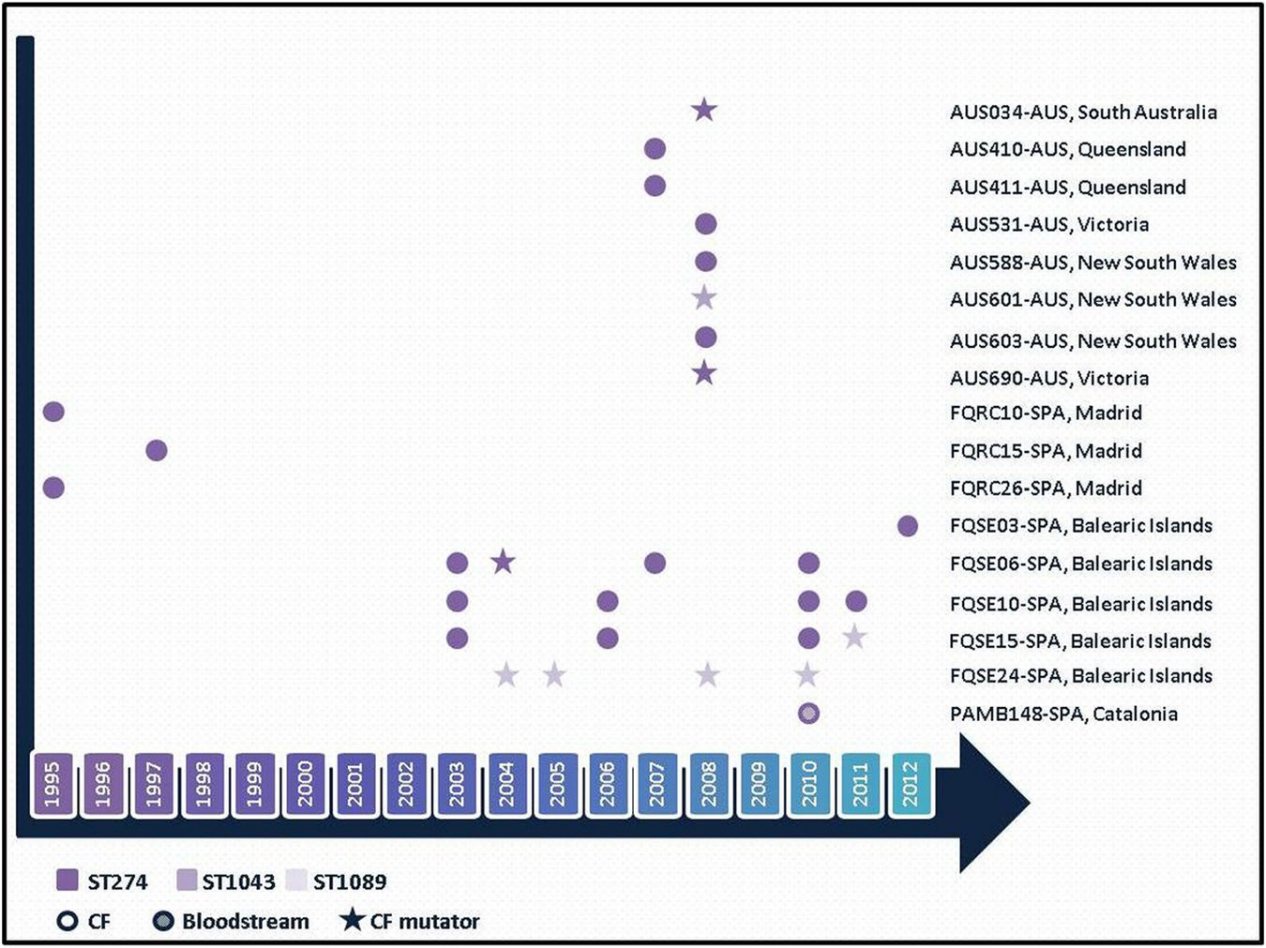
CC274 *P*. *aeruginosa* collection. Sampling time from the 29 studied isolates can be inferred from the X axis. Isolates are labelled according to the following format: Patient identification - Country (AUS: Australia; SPA: Spain), Region.

In a previous study we detected the presence of a transmissible and persistent *P*. *aeruginosa* lineage chronically infecting up to 4 of 10 selected chronically infected CF patients attended at the reference hospital of the Balearic Islands, Spain^16^. These isolates belonged to the ST274 clonal complex (CC274), which according to the MLST database, appears to colonize CF patients worldwide (http://pubmlst.org/paeruginosa/). In this work, the whole genome of a collection of CC274 strains was obtained in order to characterize the phylogeny, and the mutational resistome evolution of this widespread clonal complex; the CC274 collection included 29 representative isolates recovered from different regions of two highly distant countries, Australia and Spain, covering an 18-year period (1995–2012) and including sequential isolates from several patients (Fig. 1).

## Results and Discussion

### Prevalence and genetic basis for hypermutation: CC274 mutome

Among the CC274 studied collection, nine isolates (31%) were mutators, belonging to six (35%) different patients, residing in both Australia (n = 3) and Spain (n = 3). Data from sequential isolates were available for the Spanish isolates: one was chronically infected with a persistent mutator lineage (FQSE24), whereas the other two harbored a mixed population of mutator and non-mutator isolates (FQSE06 and FQSE15) (Fig. 1).

In order to evaluate the genetic basis of hypermutation, complementation studies with plasmids harboring wild-type Mismatch Repair system (MMR) genes (*mutS* and *mutL*) were performed in mutator isolates from these six patients. As shown in Table 1, wild-type rifampicin resistance mutation frequencies were restored in all mutator isolates upon *mutS* or *mutL* complementation, which correlated in all cases with the presence of specific mutations in these genes, documented through whole-genome sequencing. The three Australian mutator isolates showed unique mutations in either *mutL* and *mutS*. Interestingly, all mutator isolates from the three Spanish patients were found to share the same inactivating mutation in *mutS*. On the other hand, while mutator phenotypes could be explained in all cases by specific mutations in MMR genes, the contrary was not always true, since one of the non-mutator isolates showed a missense mutation in *mutS*. Moreover, the presence of polymorphisms in other mutator genes was frequent, but showed no association with mutator phenotypes (Table 1). Overall, the prevalence and genetic basis of hypermutation in CC274 was similar to that previously documented for non-clonal CF populations^9, 10^; this study is however, to our knowledge, the first investigating the genetic basis of hypermutation from whole genome sequence data, through the analysis of the sequence of an exhaustive panel of so called mutator genes, thus designated mutome.

**Table 1:** Mutator phenotype and genetic basis of hypermutation in CC274.

Isolate ID^a^	ST	Mutator?	Complement with	Sequence variation in mutator genes (mutome)^b^									
				*ung*	*mfd*	*mutS*	*sodB*	*mutT*	*sodM*	*mutL*	*mutM*	*oxyR*	*polA*
AUS034	274	Yes	*mutL*					E236D		R631C	D61N L132P		D876E
AUS410	274	No	—				E25V						D876E
AUS411	274	No	—					E236D			D61N		D876E
AUS531	274	No	—					E236D			D61N		D876E
AUS588	274	No	—				E25V						D876E
AUS601	1043	Yes	*mutL*	S13R			E25V			P159S H288Y		F106L H219Y	D876E
AUS603	274	No	—				E25V						D876E
AUS690	274	Yes	*mutS*		Q1123H	C224R T287P		E236D			D61N		D876E
FQRC10	274	No	—					E236D			D61N		D876E
FQRC15	274	No	—					E236D			D61N		D876E
FQRC26	274	No	—					E236D			D61N		D876E
FQSE03	274	No	—			L374V		E236D			D61N		D876E
FQSE06-0403	274	No	—					E236D			D61N		D876E
FQSE06-1104	274	Yes	*mutS*			Nt814Δ4		E236D			D61N		D876E
FQSE06-0807	274	No	—					E236D			D61N		D876E
FQSE06-0610	274	No	—					E236D			D61N		D876E
FQSE10-0503	274	No	—					E236D			D61N		D876E
FQSE10-0106	274	No	—					E236D			D61N		D876E
FQSE10-0110	274	No	—					E236D			D61N		D876E
FQSE10-0111	274	No	—					E236D			D61N		D876E
FQSE15-0803	274	No	—					E236D			D61N		D876E
FQSE15-0906	274	No	—					E236D			D61N		D876E
FQSE15-0310	274	No	—					E236D			D61N		D876E
FQSE15-1110	1089	Yes	*mutS*		A868T	Nt814Δ4		E236D			D61N		D876E
FQSE24-0304	1089	Yes	*mutS*			Nt814Δ4		E236D			D61N		D876E
FQSE24-1005	1089	Yes	*mutS*			Nt814Δ4		E236D			D61N		D876E
FQSE24-0308	1089	Yes	*mutS*			Nt814Δ4		E236D			D61N		D876E
FQSE24-1010	1089	Yes	*mutS*			Nt814Δ4		E236D			D61N		D876E
PAMB148	274	No	—					E236D	L202R		D61N		D876E

^a^Isolates are labelled according to the following format: Patient identification - MMYY isolation code in the case of sequential isolates.

^b^Sequence variations respect to those of PAO1. No mutations were found in other genes associated with mutator phenotypes, including *pfpI*, *mutY*, *dnaQ*, *PA2583*, *PA2819*.*1*, *PA2819*.*2*, *radA* and *uvrD*.

### Phylogeny and interpatient dissemination of the international CC274 CF clone

Pulsed Field Gel Electrophoresis (PFGE) macrorestriction patterns indicated that all isolates were clonally related, including mutators, which were indistinguishable from non-mutators. When an UPGMA (Unweighted Pair Group Method with Arithmetic Mean) dendrogram was constructed based on PFGE patterns, all isolates from the Balearic Islands clustered together in the same branch, although patterns from one of the patients (FQSE10) were slightly different. In contrast, Australian isolates were less clonal and clustered in different branches (Supplementary Fig. S1).

Conversely, by Multi Locus Sequence Typing (MLST), two new and closely ST274-related sequence types (ST) were detected. Discrepant MLST and PFGE results were linked, directly or indirectly, to the emergence of a mutator phenotype, an event that has already been documented in the CF context^16–18^. Mutators from patients FQSE15 and FQSE24 differed from ST274 by only two point mutations in two of the MLST alleles (*acsA* and *guaA*) leading to ST1089, as previously described^16^. Nevertheless, the mutator from patient FQSE06, which indeed shared the same inactivating mutation in *mutS*, still belonged to ST274 (Table 1). On the other hand, the Australian mutator AUS601 was also determined to be a new ST (ST1043), but, in this case, a direct link of the observed PFGE-MLST discrepancy with its MMR system (MutL) deficiency was suggested, since this isolate showed two missense mutations in *mutL* (Table 1), one of them (H288Y) responsible for the generation of the new ST.

**Figure 2:**
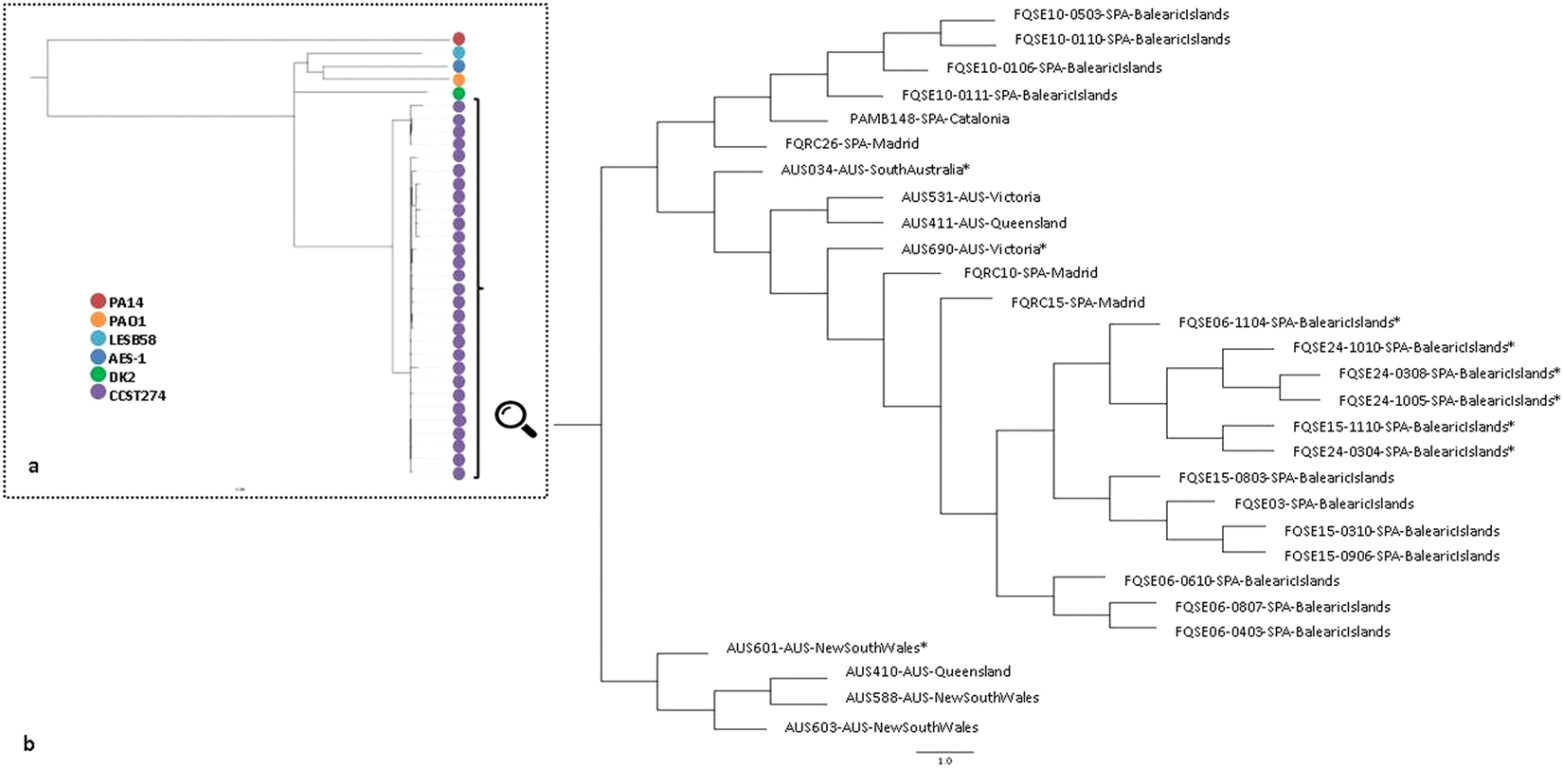
Core-genome phylogenetic reconstructions of *P*. *aeruginosa* CC274 CF clone. (**a**) Genetic relationship between CC274 and other well-recognized CF epidemic clones. (**b**) Genetic relationship between the CC274 collection isolates. Both reconstructions were made with Parsnp using default parameters. Isolates are labelled according to the following format: Patient identification - MMYY isolation code in the case of sequential isolates - Country (AUS: Australia; SPA: Spain) - Region. Mutator isolates are identified with an asterisk.

To better understand the evolutionary trajectory, success and international dissemination of CC274, whole-genome based phylogenetic analysis of all 29 isolates were performed. Previous studies have already demonstrated that almost all *P*. *aeruginosa* strains cluster into two major phylogenetic groups, one including PAO1 and the other PA14^19^. In order to determine the genetic relationship between CC274 isolates and other well-recognized CF epidemic clones, whole-genome sequence reads of all 29 isolates were *de novo* assembled and a phylogenetic tree based on core genome alignment was constructed with default parameters on Parsnp^20^. CC274 was determined to belong to the phylogenetic cluster containing strain PAO1, as well as other well-known CF epidemic clones such as LESB58, AES-1 and DK2 (Fig. 2a).

**Figure 3:**
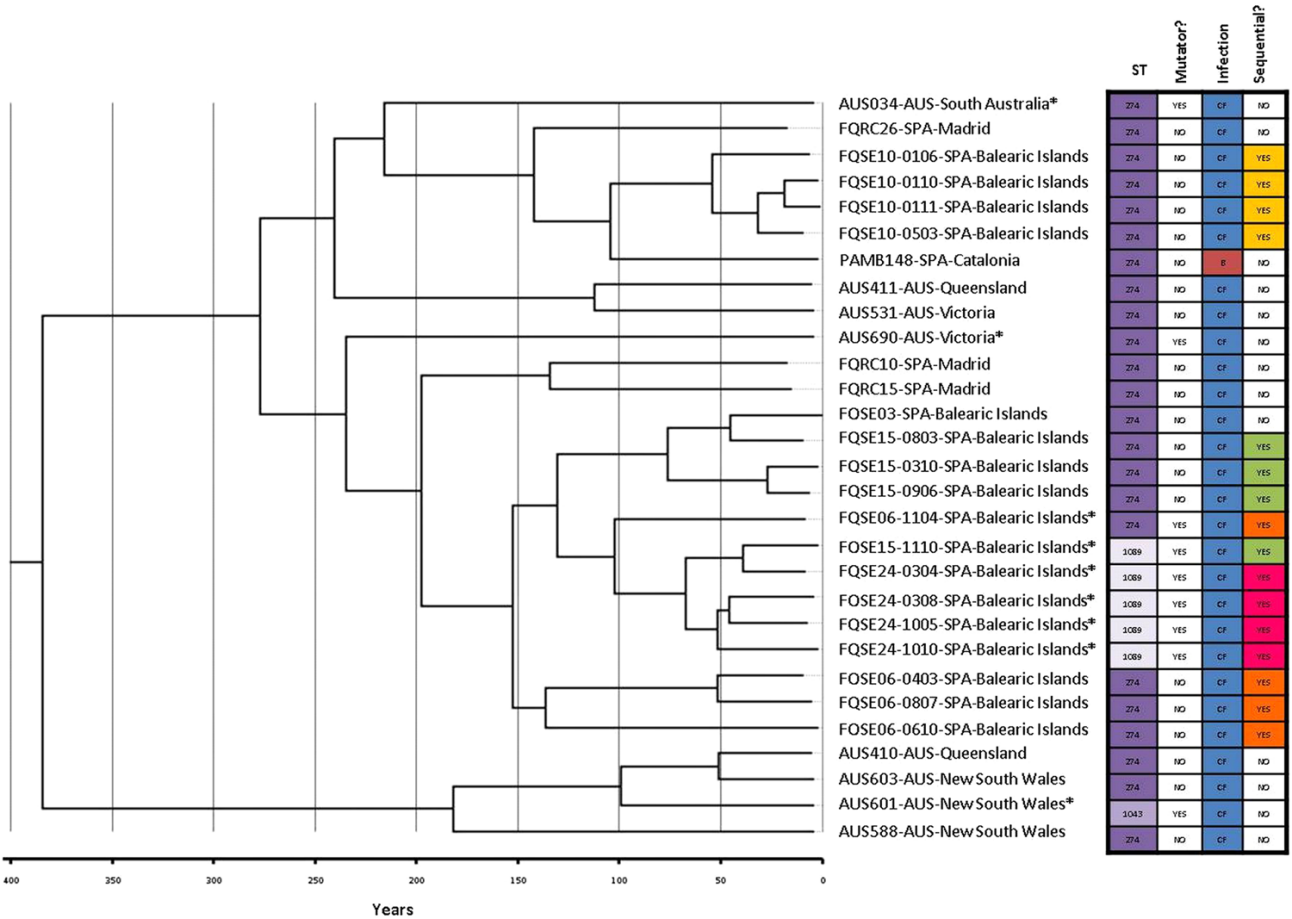
Bayesian phylogenetic reconstruction of *P*. *aeruginosa* CC274 CF clone. The tree was based on 5525 intraclonal variable positions identified by whole-genome sequencing. Divergence times of predicted ancestors and sampling dates can be inferred from the X axis taking into account that time zero corresponds to the most recent isolate (2012). The same labelling of Fig. 2 was used. Isolates characteristics are summarized at the right board, where: (CF) Cystic Fibrosis CRI and (B) Bloodstream. Sequential *P*. *aeruginosa* isolated from a same patient are indicated with the same colour.

By mapping sequence reads for each isolate against *P*. *aeruginosa* reference PAO1 strain genome, up to 16,070 common SNPs were found, as well as a total of 5,525 high-quality intraclonal SNPs, of which 2,294 were unique and thus detected in single isolates. A high degree of intraclonal diversity was observed, with SNP differences between isolates ranging from 20 to 3,256. To elucidate the phylogenetic relationship among isolates two different approaches were used. In both, core-genome and Bayesian time-based analysis, CC274 isolates grouped into two clusters, one including just four Australian isolates and a second major cluster that included all other Australian and Spanish isolates (Fig. 2b and Fig. 3). SNP differences between isolates from the different clusters ranged from 2396 to 3256 and, according to Bayesian time-based analysis, the common ancestor of CC274 was set, approximately, 380 years ago.

The major cluster further subdivided and, although both phylogenetic reconstructions did not match exactly with each other, both analyses supported that different lineages are currently coexisting with a worldwide distribution, having evolved from a common antecessor set approximately 275 years ago. SNP differences between isolates from Australia and Spain ranged from 114 to 1204, and similar results were obtained when only the Australian (min-max: 230–826) or the Spanish (min-max: 20–839) were compared, supporting no geographical barrier for lineage evolution.

Within the major cluster, all sequential isolates cultured from an individual patient clustered under the same branch with the single exception of all the Spanish isolates that exhibited a mutator phenotype which clustered together, independently of the patient involved and their ST. Along with the fact that all these mutators shared the same inactivating mutation in *mutS*, as well as many unique antibiotic resistance mutations (Supplementary Data Set S1), phylogenetic analysis clearly demonstrated that ST1089 mutators evolved from a mutator ST274 isolate and that transmission of mutators among the Spanish CF patients occurred at some time point.

Focusing on the sequential isolates, a unidirectional evolution route could not be stablished. Instead, a diversified intrapatient clone evolution that leads to a mix of genetically different sublineages coexisting in the CF respiratory airways was observed. Within a patient, minimum and maximum SNPs differences between isolates ranged from 20 to 676, which overlapped with interpatient SNPs differences, ranging from 51 to 3256 (51 to 839 for patients from the same hospital). Similar results have been reported recently by Williams *et al*. concerning the Liverpool Epidemic Strain, finding that multiple coexisting LES lineages are typically infecting CF patients and that genetic divergence between lineages within patients was greater than interpatient diversity, implying acquisition of diverse genetic populations^21^. However, another study focusing on the LES isolated from patients residing the UK and Canada showed less genetic differences, even when transoceanic isolates were compared^22^. Likewise, Yang *et al*. documented a lower genetic divergence in the DK2 epidemic clone^23^. Moreover, previous studies with other relevant and/or persistent CF clones have also reported divergent results^24–26^. A possible explanation for all these observations could be that different routes for adaptation and survival in the CF lung environment are possible and depend on the specific clonal lineages.

### CC274 resistome

Minimum inhibitory concentrations (MICs) determined for a panel of 11 antipseudomonal agents are shown in Table 2. Resistance rates were lowest for colistin (3.4%), distantly followed by ceftazidime and piperacillin-tazobactam (13.8%). In contrast, resistance to cefepime, aztreonam, imipenem, amikacin and ciprofloxacin was observed in 44.8 to 62% of the isolates. Remarkably, 17.2% of the isolates were resistant to the new combination ceftolozane-tazobactam. As shown, antibiotic resistance was more frequent among mutators, and in Australian isolates in comparison with those from Spain. In fact, all 9 mutator isolates were classified as MDR, as compared to only 3 of 20 non-mutators. Moreover, one of the Australian mutator isolates met the pan-drug resistant (PDR) definition^27^.

**Table 2:** Antibiotic susceptibility profile and main antibiotic resistance related mutations detected among CC274 isolates.

Isolate ID^a^	Antibiotic resistance profile (MIC values)^b^											Hyperexpression?			Main antibiotic resistance mutations encountered^c^
	TZ (≤8)	PM (≤8)	AT (≤1)	PPT (≤16)	C/T (≤4)	IP (≤4)	MP (≤2)	TO (≤4)	AK (≤8)	CI (≤0.5)	CO (≤2)	AmpC	MexAB	MexXY	
AUS034*	> 256	> 256	> 256	> 256	16	> 32	> 32	6	> 256	1.5	> 256	+	−	+	*gyrB* (R441L), *mexR* (R85H), *mexA* (M1*), *mexB* (F178S, M555I), *oprD*(E264*), *phoQ*(E266*), *parR* (M59I), *mexY*(V1000L), *mexZ*(Nt334Δ13), *fusA2*(P329L), PA2489(R12L, A244T), *mexS* (P254Q), *mexT*(L157M), PBP4(W350R), *capD* (I7M, S51G), *gyrA*(T83I), *mexK* (S426G), *mpl* (Nt112ins1, V124G), *fusA1* (V93A, P554L, D588G), *rpoB* (D831G, D964G), *mexW* (A627V, Q771P), PBP3 (P527T, G63S)
AUS410	4	24	1	12	4	> 32	> 32	64	> 256	1	0.38	−	−	+	*gyrB* (S466F), *mexB*(M552T), *oprD* (Nt583Δ1), *lasR* (A50V, D73G), *sucC* (V44G, A384V), *oprF* (Nt574Δ31), *mexY* (V32A), *mexZ* (Q164*), *mexT* (D327Y), *mexE* (F7Y), *mpl* (D168Y), PA2489(A125T, G185S, P260S), *capD*(I7M, S51G), *fusA1*(P618L), *rpoC*(E386K), *mexW*(Q511R), PBP3(G216S), *pagL*(Nt286Δ1), *amgS*(S64L)
AUS411	> 256	> 256	> 256	> 256	6	> 32	> 32	> 256	> 256	0.38	0.25	−	−	+	*gyrB* (S466F), *mexB* (Q104E, F246C, L376V), *phoQ* (H248P), *lasR* (D73G), *parS* (D381E, T163N), *sucC* (C261G), *mexY* (D201A, G287A), PA2489 (R12L, A244T), *fusA2* (I640L), mexE (V104G), *htpX* (Nt683Δ5), *mexK* (S426G), *capD* (I7M), *fusA1* (K504E), *rpoC* (N690S), *mexW* (A627V,Q771P), PBP3 (Q372P), *pagL* (N159D)
AUS531	3	3	4	12	1	2	0.75	1	6	0.125	1	−	−	−	PA2489 (R12L, A244T), *capD* (I7M, S51G), *mexW* (A627V, Q771P)
AUS588	2	8	3	8	1	1	0.75	1	8	0.125	0.75	−	−	−	PA2489 (A125T, G185S, P260S), *mexE* (F7Y, V276M), *capD* (I7M), *mexW* (Q511R)
AUS601*	> 256	> 256	> 256	1	3	> 32	> 32	24	> 256	16	0.25	−	−	+	*mexB* (M552T), *oprD* (Nt1044ins4), *phoQ* (K234N, T315A), *lasR* (A50V), *sucC* (T102I, A384V), *mexY* (V32A), *mexZ* (Q164*), *fusA2* (S445*), *mexT*(D327Y), *mexE*(F7Y), *ftsK* (A152V), PA2489 (A125T, G185S, P260S), *capD* (S51G), *gyrA* (T83I), *mpl* (G113D), *fusA1* (P618L), *rpoC* (E386K), *mexW* (Q511R), PBP3 (R504C), *pagL* (E163G), *pmrB* (L31P), *amgR* (E204D)
AUS603	6	8	24	2	1.5	> 32	8	1	8	0.25	1.5	+	−	+	*mexB* (M552T), *lasR* (A50V, D73G), *sucC* (V44G, A384V), *mexY* (V32A), *mexZ* (Q164*), *mexT* (D327Y), *mexE* (F7Y), PA2489 (A125T, G185S, P260S), PBP4 (S315G), *opmE* (E204D), *capD* (I7M, Nt1438Δ1), *mpl* (Nt112ins1, Nt1317Δ1), *fusA1* (P618L), *mexW* (Q511R)
AUS690*	6	12	0.75	3	6	4	2	24	> 256	12	0.125	−	+	+	*gyrB* (Q467R), *mexR* (H133P), *mexB* (Nt712Δ1), *phoP* (T221I), *lasR* (T178I), *parS* (L10P), *oprF* (K250R), *mexY* (G402S, A850T), *mexZ* (Nt529Δ1), PA2489 (R12L, A244T), *fusA2* (L104P, Nt889Δ1), *htpX* (G187D), *capD* (I7M, S51G), *gyrA* (T83A, T325I), *mexK* (G487E), *mexH* (Nt1086ins1), *fusA1* (Y552C, T671I), *rpoC* (E136G, D616G, V808L), *rpoB* (F1046S), *mexW* (A627V, Q771P), *pagL* (P158L), *pmrB* (F124L), *amgS* (R188C), *parE* (P438S)
FQRC10	2	2	4	12	1	1.5	1	1	8	0.094	0.5	−	−	−	PA2489 (R12L, A244T), *capD* (I7M, S51G), *mexH* (D356N), *mexW* (A627V, Q771P)
FQRC15	1	0.75	6	6	1	1.5	1	0.75	8	0.19	1	−	−	−	PA2489 (R12L, A244T), *capD* (I7M), *mexW* (A627V, Q771P)
FQRC26	4	6	24	24	1	0.25	1.5	1	6	1.5	0.38	−	+	−	*mexY* (V875M), *mexT* (R164H), PA2489 (R12L, A244T), *capD* (I7M, S51G), *gyrA*(Q106L), *mexW* (A627V, Q771P)
FQSE03	3	8	0.5	2	1.5	2	0.38	1	6	3	0.25	−	−	+	*mexA* (L338P), *lasR* (P117G), *mexZ* (A144V), PA2489 (R12L, A244T), *capD* (I7M, S51G), *gyrA* (D87N), *mexW* (A627V, Q771P)
FQSE06-0403	0.75	2	0.25	4	0.38	1	0.5	24	16	0.19	0.19	−	−	+	*mexA* (L338P), *lasR* (P117G), *mexY* (G287A), *mexZ* (S9P), PA2489 (R12L, A244T), *mpl* (S257L), *capD* (I7M, S51G), *fusA1* (Y552C, T671I), *mexW* (A627V, Q771P), PBP3 (P215L), *amgR* (A8V)
FQSE06-1104*	0.38	1	0.094	0.38	0.38	6	0.19	1	24	0.75	2	−	−	+	*mexA* (L338P), *lasR* (P117G), *mexZ* (A194P), PA2489 (R12L, A244T), *fusA2* (N236S, N561S), *capD* (I7M, S51G), *gyrA* (D87G), *mexK* (Q585*), *rpoB* (Y583C), *mexW* (A627V, Q771P), *pmrB* (V185I, G221D, R287Q), PBP1A (E161G), *amgR* (A8V)
FQSE06-0807	4	8	0.75	4	2	1.5	0.75	24	>256	0.5	1	−	−	+	*mexA* (L338P), *lasR* (P117G), *mexY* (G287A), *mexZ* (S9P), *mexT* (P270Q), PA2489 (R12L, A244T), *mpl* (S257L), *capD* (I7M, S51G), *fusA1* (N482S, Y552C, T671I), *mexW* (A627V, Q771P), PBP3 (P215L), *amgR* (A8V)
FQSE06-0610	4	24	0.75	8	1.5	1	0.25	1.5	24	0.75	0.19	−	−	+	*mexA* (L338P), *lasR* (P117G), *mexZ* (Nt290Δ11), PA2489 (R12L, A244T), *mexW* (A627V, Q771P), *capD* (I7M, S51G), *amgR* (A8V)
FQSE10-0503	1.5	12	4	4	1.5	1	0.25	0.75	8	0.25	0.25	−	−	+	*mexY* (V875M, N1036S), *mexZ* (IS), PA2489 (R12L, A244T), *ftsK* (A38T), *nalD* (Nt459Δ13), *mexW* (A627V, Q771P), *capD* (I7M, S51G)
FQSE10-0106	0.75	3	0.125	0.75	0.5	0.38	0.032	0.75	4	0.38	1.5	−	−	+	*mexB* (L738P), *mexY* (V875M, N1036S), *mexZ* (IS), PA2489 (R12L, A244T), *ftsK*(A38T), *capD* (S51G), *nalD* (Nt396Δ2), *mexW* (A627V, Q771P), *nfxB* (*188ext)
FQSE10-0110	3	8	16	8	2	1	0.125	0.75	4	0.75	0.5	−	+	+	*mexY* (V875M, N1036S), *mexZ* (IS), PA2489 (R12L, A244T), *ftsK* (A38T), *rpoB* (D659E, E904K), *mexW* (A627V, Q771P), *pmrB* (R287Q)
FQSE10-0111	3	16	12	12	8	1.5	1	1	12	0.38	0.38	−	−	+	*mexY* (V875M, N1036S), *mexZ* (IS), PA2489 (R12L, A244T), *ftsK* (A38T, D54Y), *capD* (S51G), *mexW* (A627V, Q771P)
FQSE15-0803	2	12	0.38	4	1.5	6	1	1	12	0.19	0.25	−	−	+	*mexA* (L338P), *lasR* (P117G), *mexZ* (A144V), PA2489 (R12L, A244T), *capD* (I7M, S51G), *pmrB* (E213D), *mexW* (A627V, Q771P), *amgR* (A8V)
FQSE15-0906	0.75	6	0.38	2	1	1	0.047	1.5	12	0.38	0.75	−	−	+	*mexA* (L338P), *lasR* (P117G), *mexZ* (A144V), *mexS* (Nt848Δ2), *mexT* (Nt534Δ17), PA2489 (R12L, A244T), *capD* (I7M, S51G), *mexK* (S426G), *mexW* (A627V, Q771P), *amgR* (A8V)
FQSE15-0310	1	4	1	1	1	12	0.19	1	8	0.38	0.25	−	−	+	*mexA* (L338P), *lasR* (P117G), *mexZ* (A144V), *mexS* (Nt848Δ2), *mexT* (Nt534Δ17), PA2489 (R12L, A244T), *capD* (I7M, S51G), *mexK* (P834S), *mpl* (Nt1266Δ1), *rpoC* (Nt1181Δ3), *mexW* (A627V, Q771P), *amgR* (A8V)
FQSE15-1110*	8	24	6	4	1	>32	>32	1	16	1	0.25	−	−	+	*gyrB* (S466F), *mexA* (N71S, D235G), *mexB* (L376V), *oprD* (V67*), *lasR* (P117G), *mexY* (Y355H), *mexZ* (A194P), galU (P123L), PA2050 (G90R, Q161R), PA2489 (R12L, A244T), *fusA2* (N236S, N561S), *htpX* (A141T), *capD* (I7M, S51G), *fusA1* (K430E), *rpoC* (V693A), *mexW* (A627V, Q771P), *pmrB* (R287Q), PBP1A (E161G), *amgS* (D267N), *amgR* (A8V)
FQSE24-0304*	2	24	0.38	8	1	>32	>32	2	24	6	0.38	−	−	+	*gyrB* (S466F), *mexA* (L338P), *oprD* (V67*), *lasR* (P117G), *mexY* (Y355H), *mexZ* (A194P), *galU* (P123L), PA2050 (G90R, Q161R), PA2489 (R12L, A244T), *fusA2* (N236S, N561S), *opmE* (D421G), *capD* (I7M, S51G), *fusA1* (K430E), *rpoC* (V693A), *mexW* (A627V, Q771P), *pmrB* (R287Q), PBP1A (E161G), *amgR* (A8V)
FQSE24-1005*	1	16	0.38	2	1.5	>32	8	3	16	6	1	−	−	+	*gyrB* (S466F), *oprD* (V67*), *lasR* (P117G), *mexY* (Y355H), *mexZ* (A194P), *galU* (P123L), PA2050 (G90R, Q161R), *fusA2* (N236S, N561S), PA2489 (R12L, A244T), *fusA1* (K430E), *rpoC*(V693A), *mexW* (A627V, Q771P), *pmrB* (R287Q), PBP1A (E161G, R407S), *amgR* (A8V)
FQSE24-0308*	1	8	0.25	0.75	1.5	>32	0.25	2	16	4	1	−	−	+	*gyrB* (S466F), *oprD* (V67*), *lasR* (P117G), *mexY* (Y355H), *mexZ* (A194P), *galU* (P123L), PA2050 (G90R, Q161R), *fusA2* (N236S, N561S), PA2489 (R12L, A244T), *capD* (I7M, S51G), *fusA1* (K430E), *rpoC* (V693A), *mexW* (A627V, Q771P), *pmrB* (R287Q), PBP1A (E161G), *amgS* (T92A), *amgR*(A8V)
FQSE24-1010*	1	8	1	1	1	>32	4	4	64	4	0.38	−	−	+	*gyrB* (S466F), *mexA* (L338P), *oprD* (V67*), *lasR* (P117G), *mexY* (Y355H), *mexZ* (A194P), *galU* (P123L), PA2050 (G90R, P97L, Q161R), PA2489 (R12L, A244T), *fusA2* (N236S, N561S), *opmE* (L400P, D421G), *mexH* (V221I), *capD* (I7M, S51G, A165V), *fusA1* (K430E), *rpoC*(V693A), *mexW* (A627V, Q771P), PBP3 (G216S), *pmrB* (R287Q), PBP1A (E161G), *amgS* (A13V), *amgR* (A8V)
PAMB148	>256	64	>256	>256	6	1.5	0.75	1.5	16	0.064	0.5	+	−	−	PA2489 (R12L, A244T), *capD* (I7M, S51G), *mexY* (V875M, N1036S), *mexW* (A627V, Q771P), *ampD* (P41L)
% I + R	13.8	44.8	48.3	13.8	17.2	44.8	27.6	24.1	62.1	48.3	3.4				

^a^Isolates are labelled according to the following format: Patient identification - MMYY isolation code in the case of sequential isolates. Mutators isolates are identified with and asterisk.

^b^Minimal Inhibitory Concentration (MIC) values were determined by grading MIC testing for the following antimicrobial agents: ceftazidime (TZ); cefepime (PM); aztreonam (AT); piperacillin-tazobactam (PPT); cefotolozane-tazobactam (C/T); imipenem (IP); meropenem (MP); tobramycin (TO); amikacin (AK); ciprofloxacin (CI) and colistin (CO). Clinical breakpoints established by EUCAST v7.0 for each antibiotic are shown in brackets.

^c^The main antibiotic resistance related mutations documented for each isolate are shown. For this purpose, the full list of mutations in the 164 genes studied (available in Supplementary Data Set S1) was refined to include only those more likely to be involved in the resistance phenotypes, by including: (i) mutations with known effect on resistance according to published evidence (ii) mutations for which our experimental evidence crosslinks resistance phenotypes and genotypes (*e*.*g*. mutations in genes involved in AmpC, efflux or OprD regulation and β-lactam resistance phenotypes are crosslinked by integrating the analysis of the expression of *ampC*, efflux pumps genes and *oprD* and/or (ii) mutations in genes found to be under high evolutionary pressure (those with at least 3 different mutational events documented).

**Figure 4:**
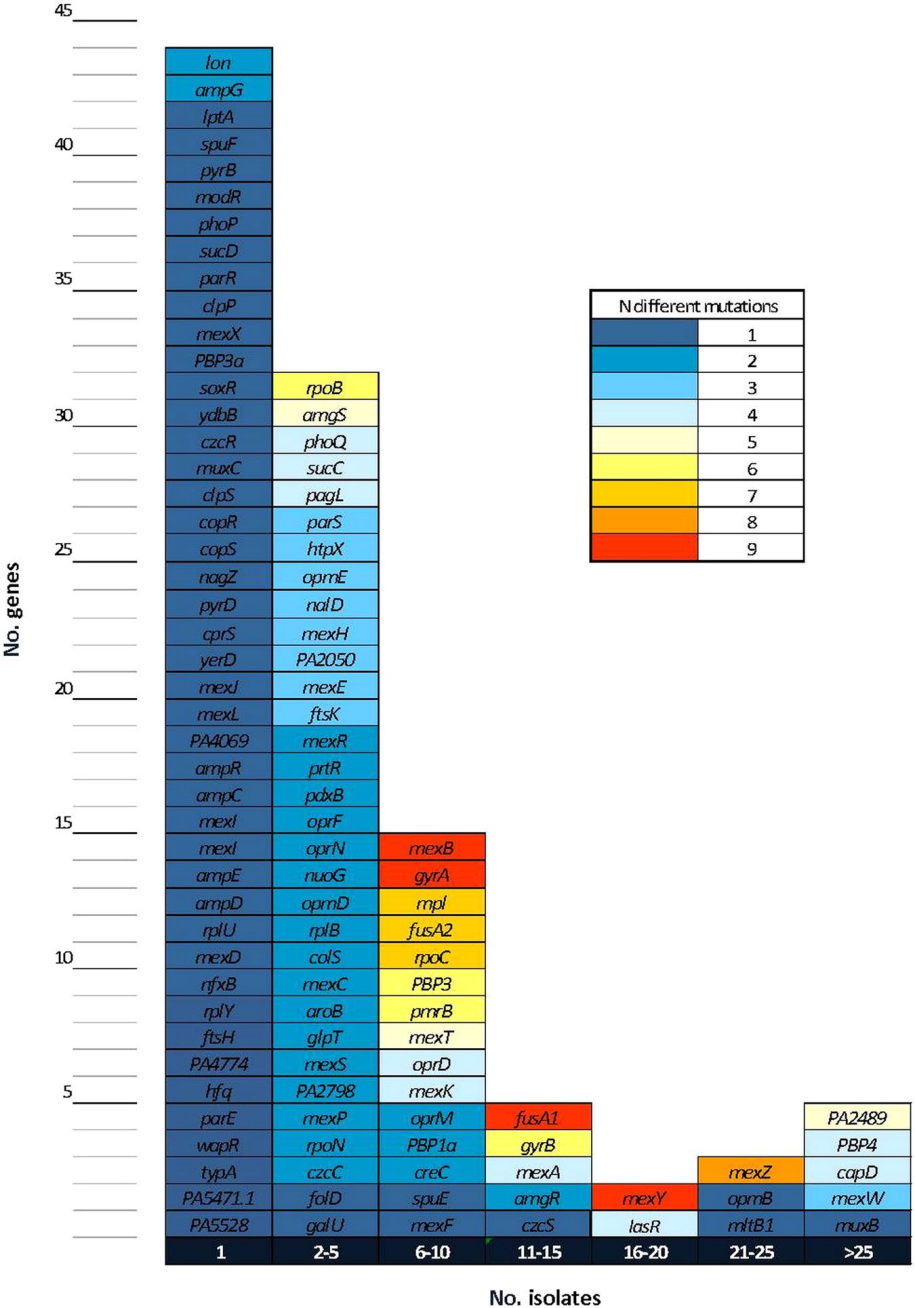
Distribution of mutations among the CC274 collection. Mutations encountered among the 164 antibiotic resistance related genes are represented, synonymous and common non-synonymous mutations have been excluded.

The presence of horizontally acquired resistance determinants was explored in the whole-genome sequences using the ResFinder tool^28^. None of the 29 isolates harbored any horizontally acquired genes encoding resistance determinants, thus indicating that the observed antibiotic resistance profiles reflected the accumulation of mutations within the chromosomal genes. The complete list of antibiotic resistance related genes investigated (n = 164) as well as all missense and non-sense mutations encountered for each of the isolates studied are reported in the Supplementary Data Set S1. Up to 127 (77.4%) of the 164 studied genes showed non-synonymous mutations in at least one of the isolates studied. Moreover, after discarding non-synonymous mutations present in all isolates (and thus considered intrinsic CC274 polymorphisms), this figure only decreased to 106 (64.6%). Figure 4 shows the number and distribution of mutations among the 164 antibiotic resistance related genes studied in the CC274 collection. Seventy-three (68.9%) of these genes showed no more than two different mutational events being 44 of them mutated in unique isolates. In contrast, 33 (31.1%) genes appeared to be under high evolutionary pressure showing evidence of at least 3 different mutational events. Particularly noteworthy among them were *mexB* or *mexY*, (coding for efflux pumps proteins), *mexZ* (the main MexXY repressor), *gyrA* (which codes for DNA gyrase subunit A) and *fusA1* (coding for the elongation factor G).

The main antibiotic resistance related mutations documented are listed in Table 2 along with the susceptibility profiles for each of the isolates. For this purpose, the full list of mutations in the 164 genes studied (available in Supplementary Data Set S1) was refined to include only those more likely to be involved in the resistance phenotypes, by including: (i) mutations with known effect on resistance according to published evidence, (ii) mutations for which our experimental evidence crosslinks resistance phenotypes and genotypes (*e*.*g*. mutations in genes involved in AmpC, efflux or OprD regulation and β-lactam resistance phenotypes are crosslinked by integrating the analysis of the expression of *ampC*, efflux pumps genes and *oprD*) and/or (ii) mutations in genes found to be under high evolutionary pressure (those with at least 3 different mutational events documented). As shown in Table 2, overall, the number of mutations was much higher (unpaired T test p < 0.0001) in mutator (19.2 ± 3.1) than in non-mutator isolates (6.7 ± 3.1). This is consistent with the much higher antimicrobial resistance of mutators, documented in this and previous works^8, 29^. However, some Australian (*e*.*g*. AUS410 or AUS411) non-mutator isolates also presented a high number of mutations, perhaps indicating that under a high antibiotic pressure in long-term CRI, MDR profiles may emerge even in the absence of mutator phenotypes. Unique mutations detected in specific genes support phylogeny reconstructions (see above Fig. 2b and Fig. 3). Moreover, they can be very useful to track interpatient transmission, considering that specific mutations detected in multiple isolates, especially within professional antibiotic resistance genes such as *gyrB*, *oprD*, *mexY*, *creC*, *mexZ* or *fusA2*, are unlikely to have occurred independently in different environments. Moreover, the analysis of these mutations can help to understand the basis for the intrapatient diversification and coexistence of multiple lineages in the CF respiratory tract.

**Figure 5:**
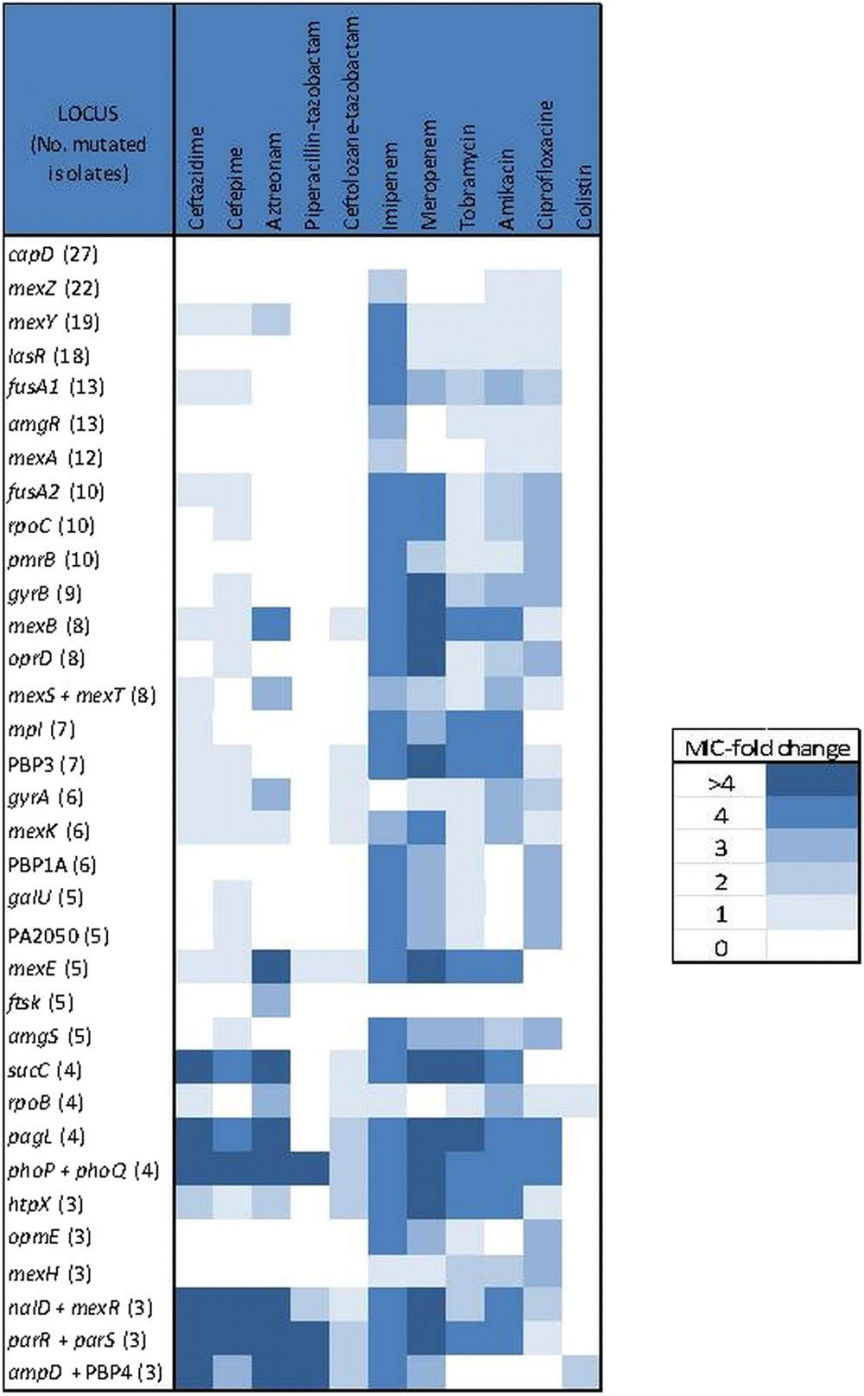
MIC-fold change for each antibiotic tested between isolates mutated or not mutated in a specific gene. To evaluate the implication of the presence of mutations in the main genes possibly related with antibiotic resistance the median MIC for both groups were calculated and compared, results are expressed in MIC-fold change. PA2489, *mexW*, *oprF*, *parE* and *nfxB* were excluded since the number of mutated isolates were < 3. Some genes were grouped (e.g. *ampD* and *dacB* (PBP4) or *nalD* and *mexR*) according to their well-established effects on resistance (e.g. AmpC or MexAB-OprM overexpression, respectively).

To gain insights into the effect on the antibiotic resistance profiles of mutations listed in Table 2, the median MIC of isolates harboring mutations or not in a specific gene were compared and results are summarized in Fig. 5. Overall, it should be noted that colistin MICs as well as the MICs for the antibiotic combinations piperacillin-tazobactam and ceftolozane-tazobactam were barely affected, whilst carbapenems, aminoglycosides and quinolones MICs are affected by the presence of mutations in many of the selected genes. Apparently, the presence of mutations in some genes such as *capD* (also known as *wbpM*), a gene coding for a protein implicated in O-antigen biosynthesis and previously related with aminoglycoside resistance, or *ftsK*, which codes for a cellular division protein, were not related with an increase in resistance for any antibiotic. Conversely, the presence of mutations in 22 of the genes was shown to produce at least a 2-fold MIC increase for at least 3 different classes of antibiotics. Renowned resistance genes, such as *gyrA*, *gyrB*, *ampD*, *dacB* (PBP4) or *oprD*, are within this list of 22 genes but, particularly interesting is the presence of not so well-recognized antibiotic resistance related genes such as *fusA1* and *fusA2*, both coding for elongation factor G, or *rpoC*, which codes the β-chain of a DNA-directed RNA polymerase. Mutations in genes coding for two-component regulatory systems, as PhoPQ or ParRS, also require a special mention as mutated isolates showed a strong impact in their MICs for many of the antibiotics tested.

The presence of unique mutations in certain well-known antibiotic resistance genes, such as *dacB* (PBP4) was observed to increase β-lactam resistance, but it should be noted that mutations within a specific gene did not always correlate or lead to the expected effect on antibiotic resistance (e.g. *pmrB* or *phoP-phoQ* mutated isolates did not exhibit a higher colistin MIC). Likewise, several mutations (e.g. *mexZ*, *gyrB* or *oprD*) were associated to extended unexpected antibiotic resistance profiles, perhaps suggesting the co-selection of different resistance mutations during *P*. *aeruginosa* evolution in CF CRI. A detailed analysis of the mutational resistome for each class of antibiotics is provided below and in the Supplementary Data Set S1.

### β-lactam resistome

Overproduction of the chromosomally encoded cephalosporinase AmpC is the primary pathway for developing resistance to the antipseudomonal β-lactams, and it is driven by the selection of mutations in peptidoglycan-recycling genes (*ampD*, *dacB* and *ampR*)^30, 31^. Just three isolates (AUS034, AUS603 and PAMB148) of the CC274 collection were demonstrated to overproduce AmpC (Table 2). By contrast, at the genomic level, almost all isolates (26/29) contained some variation within *dacB* which codes for the penicillin-binding protein PBP4 (Supplementary Data Set S1-Betalactams). Crosslinking phenotypic and genotypic results through *ampC* expression data, suggested that most observed *dacB* allele variations were, in fact, ancestral polymorphisms not involved in antibiotic resistance. However, AmpC overproduction in the two CF isolates was explained by the presence of specific mutations in *dacB* (S315G or W350R) and by an *ampD* (P41L) mutation in the case of the bloodstream infection isolate PAMB148 (Table 2). Whilst *ampC* overexpression in isolates AUS034 and PAMB148 correlated well with ceftazidime and piperacillin-tazobactam resistance, this was not the case for isolate AUS603 which was documented to be susceptible to these antibiotics. However, unexpected AUS603 β-lactam susceptibility could be explained by the presence of chromosomal mutations whose effects eventually compensate the expected increase in β-lactam resistance. Indeed, this isolate showed a non-sense mutation in OprM (Q93X), the outer membrane protein of the constitutive MexAB efflux pump, which is well known to play a major role in intrinsic β-lactam resistance.

In addition, *P*. *aeruginosa* may eventually develop β-lactam resistance by acquiring mutations within their macromolecular targets: the essential penicillin-binding proteins (PBPs). While some mutations without apparent effect on resistance were detected in genes coding for PBP1 and PBP3a, the main mutational resistance target among PBPs was found to be PBP3, an essential high molecular class B PBP with transpeptidase activity, in agreement with recent data from CF patients^32^ and *in vitro* studies^33^. Indeed, we documented that PBP3 mutations had often occurred (7/29 isolates) among the CC274 collection (Supplementary Data Set S1-Betalactams). Nevertheless, β-lactam resistance contribution of each derived *ftsI* (PBP3) allele, if any, depends on the specific point mutation encountered. Missense mutations within the PBP3 (R504C and Q372P) were apparently the cause of β-lactam resistance in isolates AUS601 and AUS411, since they do not hyperproduce AmpC. Although these mutations are not located in the PBP3 active site, both are very close to two loop regions (residues 332–338 and 526–533) which play an important role in substrate recognition^34^. In fact, PBP3 mutations in residue 504 (R504C, R504H) have been recently described *in vitro* ^33^ and among isolates from widespread nosocomial *P*. *aeruginosa* clones^35, 36^. Likewise, the P527T mutation of AUS034 likely contributes, together with the overexpression of AmpC, to the very high-level β-lactam resistance of this isolate, including the new antipseudomonal combination ceftolozane-tazobactam. On the other hand, the P215L and G216S mutations were apparently not linked with phenotypic resistance, in agreement with the fact that residues 215 and 216 are not implicated in the formation and stabilization of the inactivating complex β-lactam-PBP3^34^.

Obtained data also demonstrated that the constitutive efflux pump MexAB-OprM is under strong mutational pressure during CF CRI, frequently including inactivating mutations, which correlates with previous investigations that pointed out that this efflux pump is dispensable and, therefore, tends to be lost or inactivated in favor of MexXY-OprM hyperproduction in CF *P*. *aeruginosa* subpopulations^37^. Our data also support this hypothesis, as just 3 isolates showed mutations in regulators leading to MexAB-OprM overexpression, whereas up to 23 isolates hyperproduced the efflux-pump MexXY-OprM (Supplementary Data Set S1 -Betalactams). Moreover, many of the isolates showed some degree of hypersusceptibiltiy to aztreonam (substrate of MexAB-OprM) in favor of an increased MIC of cefepime (substrate of MexXY-OprM) (Supplementary Data Set S1- Betalactams).

### Carbapenem resistome

Imipenem and meropenem resistance correlated in all but two isolates with the presence of non-sense mutations affecting the outer membrane protein OprD (Table 2, Supplementary Data Set S1-Carbapenems). High-level meropenem resistance was additionally associated with the presence of PBP3 mutations, in agreement with recent *in vitro* studies showing the selection of PBP3 mutations upon meropenem exposure^33^. Remarkably, all ST1089 mutator isolates shared the same point mutation in *oprD* (V67X) as well as in *galU* (P123L), also related with carbapenem resistance, supporting interpatient transmission of this mutator lineage among CF patients attending the reference hospital of the Balearic Islands.

The expression of OprD is known to be modulated by mutations (*mexS* or *mexT*) leading to the overexpression of the efflux pump MexEF-OprN, and meropenem is a well-known substrate for the efflux pump MexAB-OprM. However, carbapenem resistant isolates AUS411 and AUS603 harboring a wild-type *oprD* allele did not overproduce neither of these two efflux pumps. Thus, the observed phenotype could be related with the presence of specific mutations within the genes coding for PBP3 (*ftsI*) and PBP4 (*dacB*), hypothesis that is current being evaluated in our laboratory.

### Aminoglycoside resistome

Intravenous antimicrobial combinations including an aminoglycoside plus a fluroroquinolone or a β-lactam antibiotic are frequently used to manage CF exacerbations. Moreover, in the last decade, tobramycin inhalation has become an important contributor to CF treatment as a means to control chronic infection as well as a first-line treatment for the eradication of early acquisition of *P*. *aeruginosa* and several aminoglycoside-based inhaled formulations are currently available. Resistance to antipseudomonal aminoglycosides is frequently attributed to the presence of acquired aminoglycoside-modifying enzymes, membrane impermeability or MexXY efflux pump overexpression^38^. Moreover, adaptive resistance, due to MexXY efflux system overexpression, to this class of antibiotics has been well documented in the CF setting in response to sublethal concentrations^39^.

Among the CC274 collection, a high proportion of the isolates (23/29) were shown to overexpress MexXY and all but one were mutated in *mexZ*, which codes for the mayor MexXY expression regulator. In agreement with recent work, which pointed out that mutation of *mexZ* is part of a strongly selected evolutionary pathway^40^, several different mutational events were encountered within this regulator. Remarkably, the same point mutation was detected among different and independent isolates, probably indicating interpatient transmission events (Table 2, Supplementary Data Set S1-Aminoglycosides). The single MexXY-overproducing isolate showing no mutations in *mexZ*, presented a unique mutation in *parS*, a gene also involved in the modulation of MexXY expression. Nevertheless, as it has been largely observed in the CF clinical setting^41^, MexXY hyperproduction *per se* cannot explain aminoglycoside resistance in the majority of the isolates. In this sense, there is growing evidence that high-level resistance is a stepwise process which arises by the accumulation of several non-enzymatic mechanisms and, moreover, novel genetic resistance determinants have been proposed^42–44^. To our knowledge, no published work has yet investigated the *in vivo* contribution to aminoglycoside resistance of these novel genetic determinants proposed, and many questions remain unresolved. Thus, our work reveals for the first time that all high-level resistant isolates hyperproduced MexXY, but also harbored additional mutations in some of these genes, especially highlighting the presence of mutations in both genes coding for elongation factor G, *fusA1* and *fusA2* (Supplementary Data Set S1 - Aminoglycosides). In fact, *fusA1* and *fusA2* have been recently demonstrated to be under high evolutionary pressure in the CF environment, which can be explained in terms of a wide aminoglycoside use in this setting^45^.

### Fluoroquinolone resistome

*P*. *aeruginosa* RND (Resistance-Nodulation-Division) efflux pumps MexAB-OprM, MexXY-OprM, MexCD-OprJ and MexEF-OprN are well-known to extrude fluoroquinolones. Nevertheless, our data suggest that the contribution of the overexpression of these efflux pumps to high-level resistance to fluoroquinolones is very limited, if any (Supplementary Data Set S1 -Fluoroquinoles). Concerning MexCD-OprJ overproduction, it has been shown that, although wild type *P*. *aeruginosa* strains generally do not express this efflux system^46^, hyperproducing mutants tend to emerge after both *in vitro* and *in vivo* fluoroquinolone exposure^33^. Moreover, there is some data suggesting that MexCD-OprJ hyperproduction could be an advantage in the CF environment^47^. Among the CC274 collection, however, just 1 isolate (FQSE10-0106), showing aa ciprofloxacin MIC below the resistance breakpoint (MIC = 0.38 mg/L), was demonstrated to hyperproduce MexCD-OprJ due to a non-sense mutation in *nfxB*.

On the other hand, our data shows that high-level fluoroquinolone resistance was associated with the presence of missense mutations in *gyrA*, *gyrB* and/or *parC* quinolone resistance-determining regions (QRDRs). Specifically, up to 9 isolates were mutated in *gyrB* QRDR and all but two harbored the same mutation (S466F), 6 showed mutations in *gyrA* QRDR (T83I, T83A, D87N, D87G and Q106L), and just one isolate was mutated in *parE* (P438S). Mutations in GyrA residues 83 and 87 are well-known to be relevant in the clinical setting and are frequently encountered in fluoroquinolone-resistant *P*. *aeruginosa* ^35, 36^, being both residues situated on helix-4. Mutations in residue 106 are in the other hand very infrequent, with only one previous reference of its existence in 1 of 335 quinolone resistant *P*. *aeruginosa* clinical strains^48^.

### Polymyxin resistome

Two component-regulatory systems as well as other genes implicated in lipopolysaccharide biosynthesis have been related with polymyxin resistance^49–51^. Several *in vitro* works have addressed the implication of the two-component regulatory systems in polymyxin resistance development, demonstrating that individual alterations in these systems are generally not sufficient to develop high-level resistance^50, 52–54^ and that individual two-component systems may not be essential for acquisition of colistin (polymyxin E) resistance in *P*. *aeruginosa* ^55^. In agreement with these *in vitro* studies, we found that many isolates were mutated in genes such as *pagL*, *phoQ* or *pmrB*, but with one exception (i.e. isolate AUS034) phenotypic resistance was not observed (Table 2, Supplementary Data Set S1-Polymyxins). For isolate AUS034, a specific non-sense mutation was detected in the two-component sensor PhoQ, as well as two other specific point mutations within *parR* and *colS*. Five additional isolates were shown to harbour mutations in more than one polymyxin-resistance related genes and showing colistin MICs from 0.125 to 2 mg/L (Supplementary Data Set S1- Polymyxins). Remarkably, none of the mutations detected in the two-component regulatory systems PmrAB and PhoPQ have been previously described in the clinical setting, reflecting an individual strain adaptation to the CF lung^36, 52, 53, 56^. Six different and independent mutational events were registered in PmrB sensor, being all but two located near the active site (H249). Moreover, Spanish mutators shared the same mutation, again reflecting the interpatient transmission of a CF-adapted mutator lineage.

Considering that colistin is widely used for the management of CF patients, the frequent documentation of mutations in genes such as *phoQ*, *pmrB* or *pagL* suggests a role in polymyxin resistance, tolerance or adaptation *in vivo*, even when phenotypic resistance is not demonstrated *in vitro*. Thus, further *in vivo* and clinical studies should be performed to decipher the impact of these mutations for the therapeutic management of CF patients.

### Concluding remarks

Emergence of international epidemic CF clonal lineages, along with the extraordinary capacity of *P*. *aeruginosa* to develop resistance to all antibiotic classes, catalyzed by frequent mutator phenotypes, severely compromises the clinical management of *P*. *aeruginosa* CF CRI. In addition to the assessment of the emerge of mutator phenotypes within an international CF clone, we analyzed for the first time the genetic basis of hypermutation from whole genome sequence data, through the analysis of the sequence of an exhaustive panel of so called mutator genes, thus designated mutome.

CC274 population structure analysis demonstrated the coexistence of two separated and divergent clonal lineages, but without evident geographical barrier. Coexistence of distinct evolved sublineages within a patient was documented, reflecting coexistence of divergent lineages within the infecting inoculum or alternatively, and less probable, multiple interpatient transmission events. More revealing is the confirmation, by both phylogenetic reconstructions and mutational resistome analysis, of interpatient transmission of mutators. Compared with classical molecular typing tools, WGS provides detailed genome fingerprints that might be essential for epidemiological studies in which prevalent and ubiquitous clonal lineages are involved. Indeed, WGS closely clustered isolates from four of the patients from the Balearic Islands, likely indicating interpatient transmission or a common source of colonization, whereas isolates from a fifth patient from the same hospital was distantly related.

We have documented at whole genome level the extraordinary capacity of *P*. *aeruginosa* to acquire resistance by mutational events, evidencing the emergence of mutations in over 100 genes related to antibiotic resistance during the evolution of a CF epidemic clone. Moreover, our results confirm that the evolution of *P*. *aeruginosa* resistome is greatly enhanced when mutator phenotypes are selected. However, the difficulty for correlating genotypic with phenotypic variation (due to random drift mutations among other causes) has been a hallmark of WGS approaches. To minimize this limitation, the full list of mutations in the 164 genes studied was refined to include only those more likely to be involved in the resistance phenotypes. While the presence of classical mutational mechanisms, such as the overexpression of the β-lactamase AmpC, the inactivation of the carbapenem porin OprD, or QRDR mutations, was confirmed in a number of isolates and correlated with the resistance phenotypes, our results also provided evidence for the existence and important role of less expected resistance mutations and their phenotypes. Among them, PBP3 mutations, shaping up β-lactam resistance are particularly noteworthy. Likewise, our work, as previously others, denote the very high selective pressure for *mexZ* mutations, leading to the overexpression of MexXY, associated with aminoglycoside resistance. However, we show for the first time that high-level aminoglycoside resistance in CF is driven by the acquisition of additional mutations, particularly those in *fusA1* or *fusA2*, coding for elongation factor G. Finally, a complex repertoire of mutations in genes related to polymyxin resistance is evidenced, but with limited correlation with *in vitro* phenotypic resistance. Altogether, our results provide valuable information for understanding the evolution and dynamics of the mutational resistome of *P*. *aeruginosa* CF clones and it is correlation with resistance phenotypes, which might be useful for guiding new diagnostic tools and therapeutic strategies in CRI.

## Material and Methods

### *P*. *aeruginosa* CC274 collection and susceptibility testing

The CC274 collection included 29 isolates: 28 recovered from 18 CF patients from Australia and Spain and 1 blood culture isolate from a Spanish non-CF patient, covering up to an 18-year period from 1995 to 2012. All isolates had been previously classified within the CC274 (sharing at least 5 alleles with ST274) based on MLST using available protocols and databases (http://pubmlst.org/paeruginosa/). All the Australian and 4 CF Spanish isolates were single isolates recovered from patients attending clinical settings located in different geographical areas, being each area represented by at least 2 independent isolates, selected randomly from those available. In addition, we included 4 sequential *P*. *aeruginosa*, each separated by at least 6-month intervals, from each of 4 CF patients attended at the reference hospital of the Balearic Islands (Son Espases Hospital, Spain)(Fig. 1), thus representing intrapatient clone evolution. These patients were shown to be chronically colonized with this persistent strain in a previous study^16^. *P*. *aeruginosa* PAO1 strain was used as reference when needed. Minimal inhibitory concentrations (MICs) of ceftazidime, cefepime, aztreonam, piperacillin-tazobactam, ceftolozane-tazobactam, imipenem, meropenem, tobramycin, amikacin, ciprofloxacin and colistin were determined by Etest and classified according EUCAST clinical breakpoints (http://www.eucast.org/).

### Molecular typing

Clonal relatedness among isolates was evaluated by PFGE. For this purpose, bacterial DNA embedded in agarose plugs prepared as described previously was digested with SpeI. DNA separation was then performed in a contour-clamped homogeneous-electric-field DRIII apparatus (Bio-Rad, La Jolla, CA) under the following conditions: 6 V/cm^2^ for 26 h with pulse times of 5 to 40 s. DNA macrorestriction patterns were analyzed with UPGMA to infer clonal relatedness (CLIQS 1D Pro, Totallab).

### Mutant frequencies and genetic basis of hypermutation

Rifampicin (300 mg/L) resistance mutant frequencies were determined in all strains following previously established procedures^9, 10^. To explore the genetic basis for the mutator phenotypes, complementation studies were performed as described previously^9^. Briefly, plasmid pUCPMS harbouring PAO1 wild-type *mutS*, plasmid pUCPML harbouring PAO1 wild-type *mutL*, and plasmid pUCP24, a control cloning vector, were electroporated into the mutator isolates. Complementation was demonstrated by reversion of the increased rifampicin resistance mutant frequencies in two independent transformant colonies for each strain. Additionally, the genetic basis of hypermutation was investigated from whole genome sequence data, through the analysis of an exhaustive panel of so called mutator genes, thus designated mutome. Genes included within the mutome panel, selected according to available informatio^8^ were the following: PA0355/*pfpI*, PA0357/*mutY*, PA0750/*ung*, PA1816/*dnaQ*, PA3002/*mfd*, PA3620/*mutS*, PA4366/*sodB*, PA4400/*mutT*, PA4468/*sodM*, PA4609/*radA*, PA4946/*mutL*, PA5147/*mutM*, PA5344/*oxyR*, PA5443/*uvrD* and PA5493/*polA*.

### Characterization of resistance mechanisms

The levels of expression of *ampC*, *mexB*, *mexD*, *mexY*, and *mexF* were determined by real-time reverse transcription (RT)-PCR according to previously described protocols^57^. Additionally, for selected isolates, the sequences of resistance genes, such as *oprD* or mexZ was obtained by Sanger sequencing in order to confirm whole-genome sequencing data as needed. Briefly, after duplicate PCR amplification, sequencing reactions were performed with the BigDye Terminator kit (PE Applied Biosystems, Foster City, CA), and sequences were analyzed on an ABI Prism 3100 DNA sequencer (PE Applied Biosystems). The resulting sequences were then compared with that yielded by WGS technology.

### Library preparation and whole-genome sequencing

Genomic DNA was obtained by using a commercially available extraction kit (High Pure PCR template preparation kit; Roche Diagnostics). Indexed paired-end libraries were prepared with Nextera XT DNA library preparation kit (Illumina Inc, USA) and sequenced on an Illumina MiSeq® benchtop sequencer with MiSeq reagent kit v2 (Illumina Inc., USA), resulting in 250 bp paired-end reads.

### Variant calling

Previously defined and validated protocols were used with slight modifications^25, 58^. Briefly, paired-ended reads were aligned to the *P*. *aeruginosa* PAO1 reference genome (GenBank accession: NC_002516.2) with Bowtie 2 v2.2.4 (http://bowtie-bio.sourceforge.net/bowtie2/index.shtml)^59^ and, eventually, pileup and raw files were obtained by using SAMtools v0.1.16 (https://sourceforge.net/projects/samtools/files/samtools/)^60^ and PicardTools v1.140 (https://github.com/broadinstitute/picard). The Genome Analysis Toolkit (GATK) v3.4-46 (https://www.broadinstitute.org/gatk/) was used for realignment around InDels^61^. Median PAO1 coverage was 95.75% (range: 90.4–97.6%). SNPs were extracted from the raw files if they met the following criteria: a quality score (Phred-scaled probability of the samples reads being homozygous reference) of at least 50, a root-mean-square (RMS) mapping quality of at least 25 and a coverage depth of at least 3 reads; excluding all ambiguous variants. MicroInDels were extracted from the totalpileup files applying the following criteria: a quality score of at least 500, an RMS mapping quality of at least 25 and support from at least one-fifth of the covering reads. Finally, all positions in which at least one of the isolates showed some variation were manually and individually checked in all other isolates without applying any filtering.

### ***De novo*** assembly

Sequence reads from each isolate were *de novo* assembled using Velvet v1.2.10 (https://www.ebi.ac.uk/~zerbino/velvet/)^62^ with a k-mer length of 31 and the following parameters: scaffolding = no, ins_length = 500, cov_cutoff = 3, and min_contig_lgth = 500. The median size of the *de novo* assembled obtained genomes was 6.1Mbp, ranging from 5.4 to 6.6Mbp. MUMmer3 v3.23^63^ was used to align the obtained genomes against each other in order to confirm that all belong to the same clone type (genomes differing < 10,000 SNPs).

### Phylogenetic reconstructions and BEAST analysis

Core genome phylogenetic reconstructions were performed using Parsnp from the Harvest Suite package v1.2 with default parameters forcing the inclusion of all genomes and a randomly selected reference genome (flags: -c / -r!) (http://harvest.readthedocs.io/en/latest/content/parsnp.html)^20^. Bayesian analysis of divergence times was performed using BEAST v2.4.2 (http://beast2.org/)^64^. For this purpose, a nexus file including all the curated positions at which at least one of the isolates differed from the reference strain PAO1 was constructed and converted into an.xml file with BEAUTi. BEAST was run with the following user-determined settings; a lognormal relaxed molecular clock model and a general time-reversible substitution model with gamma correction^25^. Divergence times were calculated from a chain length of 50 million steps, sampled every 1,000 steps and discarding the first 5 million steps as a burn-in. The maximum clade credibility tree was generated using the TreeAnnotator program from the BEAST package and tree parameters were calculated with Tracer v1.6 (http://beast.bio.ed.ac.uk/Tracer). Both Phylogenetic reconstructions were displayed using FigTree v1.4.2 (http://tree.bio.ed.ac.uk/software/figtree/).

### Profiling of antibiotic resistance genes

SNPs and InDels for each isolate were annotated by using SnpEff software v4.2 (http://snpeff.sourceforge.net/index.html)^65^ with default options. These files were then filtered based on an exhaustive literature review^35^ that led us to obtain a set of 164 genes known to be related to chromosomal antibiotic resistance in *P*. *aeruginosa* (Supplementary Data Set S1). Additionally, we used the online tool ResFinder v2.1 (https://cge.cbs.dtu.dk//services/ResFinder/)^28^ to identify possible horizontally acquired antimicrobial resistance genes.

### Ethics statement

The study has been approved by the Research Committee from Son Espases University Hospital. All methods were performed in accordance with the relevant guidelines and regulations. Used isolates derived from frozen stocks of laboratory collections obtained from routine cultures. Patient’s information or tissue samples were not used in this study.

## Electronic supplementary material

Supplementary information.

Supplementary information
