# Supplementary material for: Evolution of the Pseudomonas aeruginosa mutational resistome in an international Cystic Fibrosis clone
Source: Sci Rep. 2017 Jul 17;7:5555. doi: 10.1038/s41598-017-05621-5 (PMC5514035; doi:10.1038/s41598-017-05621-5)
Supplement: Supplementary file 2 — Supplementary information [file 41598_2017_5621_MOESM2_ESM.pdf]

Supplementary Material

Evolution of the *Pseudomonas aeruginosa* mutational resistome in an international Cystic Fibrosis clone.

Carla López-Causapé, Lea Mette Sommer, Gabriel Cabot, Rosa Rubio, Alain A. Ocampo-Sosa, Helle Krogh Johansen, Joan Figuerola, Rafael Cantón, Timothy J. Kidd, Soeren Molin and Antonio Oliver.

Evolution of the *Pseudomonas aeruginosa* mutational resistome in an international Cystic Fibrosis clone. Carla López-Causapé, Lea Mette Sommer, Gabriel Cabot, Rosa Rubio, Alain A. Ocampo-Sosa, Helle Krogh Johansen, Joan Figuerola, Rafael Cantón, Timothy J. Kidd, Soeren Molin and Antonio Oliver.

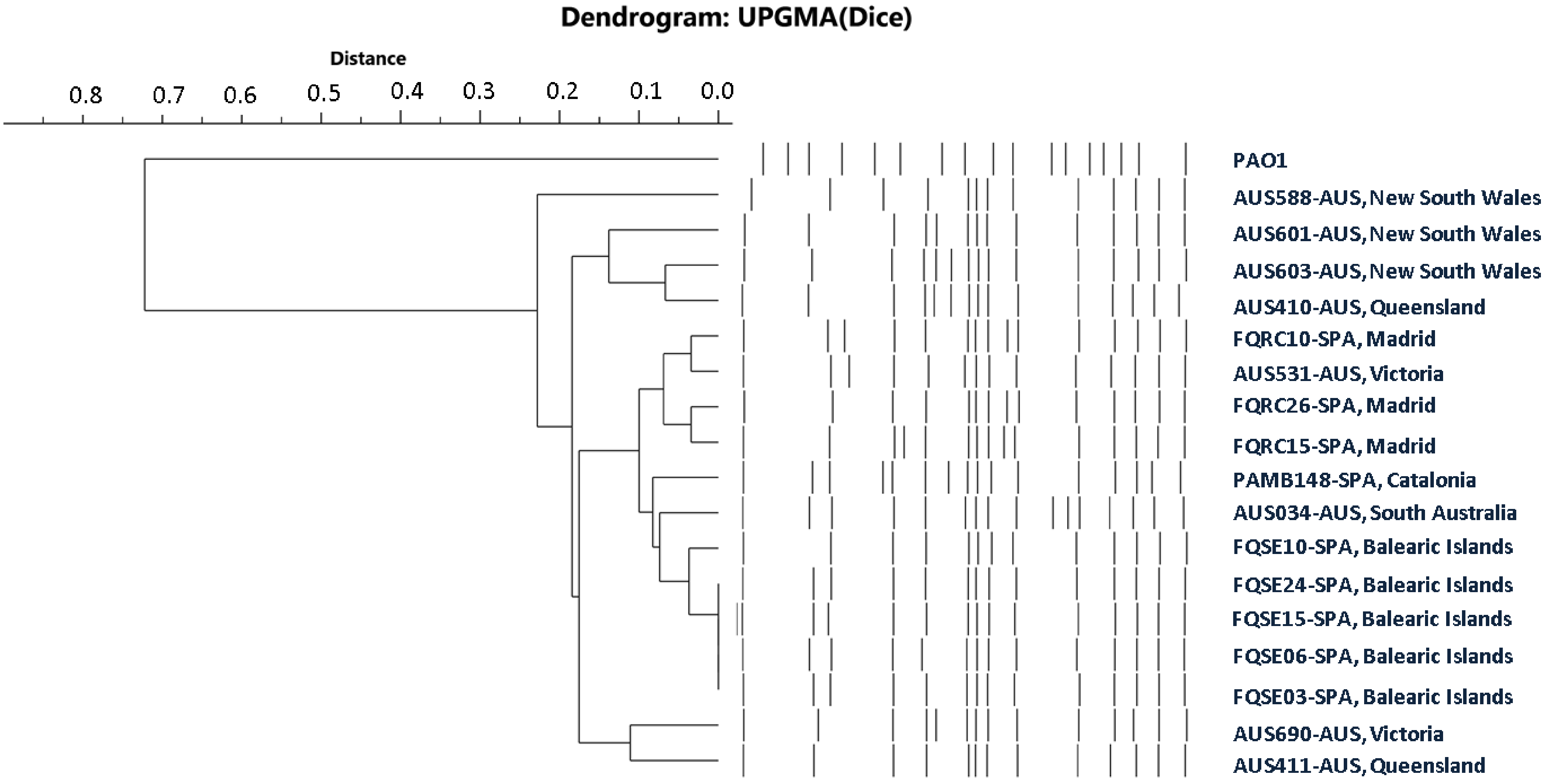

Supplementary Figure S1. UPGMA phylogenetic tree showing the relationship among the CC274 *P. aeruginosa* collection. The tree was constructed based on the DNA macrorestriction fragment patterns obtained by pulsed-field gel electrophoresis (PFGE) using SpeI restriction enzyme Isolates are labelled according to the following format: Patient identification - Country (AUS: Australia; SPA: Spain), Region.
